# Metabolic resilience of the Australasian snapper (*Chrysophrys auratus*) to marine heatwaves and hypoxia

**DOI:** 10.3389/fphys.2023.1215442

**Published:** 2023-07-17

**Authors:** Lyvia R. Bowering, Tristan J. McArley, Jules B. L. Devaux, Anthony J. R. Hickey, Neill A. Herbert

**Affiliations:** ^1^ Institute of Marine Science, University of Auckland, Leigh, New Zealand; ^2^ School of Biological Sciences, University of Auckland, Auckland, New Zealand

**Keywords:** hypoxia-tolerance, thermal-tolerance, mitochondria, respirometry, heart, fish, metabolism

## Abstract

Marine organisms are under threat from a simultaneous combination of climate change stressors, including warming sea surface temperatures (SST), marine heatwave (MHW) episodes, and hypoxic events. This study sought to investigate the impacts of these stressors on the Australasian snapper (*C. auratus*) — a finfish species of high commercial and recreational importance, from the largest snapper fishery in Aotearoa New Zealand (SNA1). A MHW scenario was simulated from 21°C (current February SST average for north-eastern New Zealand) to a future predicted level of 25°C, with the whole-animal and mitochondrial metabolic performance of snapper in response to hypoxia and elevated temperature tested after 1-, 10-, and 30-days of thermal challenge. It was hypothesised that key indicators of snapper metabolic performance would decline after 1-day of MHW stress, but that partial recovery might arise as result of thermal plasticity after chronic (e.g., 30-day) exposures. In contrast to this hypothesis, snapper performance remained high throughout the MHW: 1) Aerobic metabolic scope increased after 1-day of 25°C exposure and remained high. 2) Hypoxia tolerance, measured as the critical O_2_ pressure and O_2_ pressure where loss of equilibrium occurred, declined after 1-day of warm-acclimation, but recovered quickly with no observable difference from the 21°C control following 30-days at 25°C. 3) The performance of snapper mitochondria was also maintained, with oxidative phosphorylation respiration and proton leak flux across the inner mitochondrial membrane of the heart remaining mostly unaffected. Collectively, the results suggest that heart mitochondria displayed resilience, or plasticity, in snapper chronically exposed to 25°C. Therefore, contrary to the notion of climate change having adverse metabolic effects, future temperatures approaching 25°C may be tolerated by *C. auratus* in Northern New Zealand. Even in conjunction with supplementary hypoxia, 25°C appears to represent a metabolically optimal temperature for this species.

## Introduction

Increased and more variable sea surface temperatures (SST) and intensification of hypoxia are amongst the list of marine stressors projected to worsen with climate change (Diaz & Breitburg, 2009; Pisano et al., 2020). Marine heatwaves (MHW), defined as an extended timeframe of excessively warm water temperatures, are increasing in frequency, intensity, and persistence (Hobday et al., 2018; Oliver et al., 2018). Recent MHWs have impacted marine ecosystems (Thomsen et al., 2019; Bell et al., 2023) and fisheries (Mills et al., 2013; Pershing et al., 2015) and are thought to be of a greater ecological concern than the forecasted gradual SST increase (Frölicher et al., 2018; Frölicher & Laufkötter, 2018; Smale et al., 2019). Climate change, along with continued urban and agricultural expansion, is also expected to intensify instances of hypoxia—particularly in coastal and estuarine ecosystems—as levels of dissolved O_2_ less than <55% air saturation become more frequent (Diaz, 2001; Vaquer-Sunyer & Duarte, 2008; Diaz & Breitburg, 2009). Low dissolved O_2_, whilst commonly caused by anthropogenic activity, can also occur as a result of natural processes, such as eutrophication from coastal upwelling and from diel-cycling in shallow estuarine systems, where respiration at night creates hypoxia in the absence of photosynthesis (McNatt & Rice, 2004; Baumann et al., 2015). However, climate change stressors are not expected to occur in isolation (Boyd et al., 2015). For example, the challenge of high temperature in aquatic environments frequently co-occurs with hypoxia, as increased water temperature decreases the solubility of O_2_ in water, reduces O_2_ mixing as a result of water column stratification, and elevates the respiratory O_2_ demands of aquatic organisms (Diaz & Breitburg, 2009; Altieri & Gedan, 2015).

Aerobic metabolic performance is thought to be a key driver of thermal tolerance (Pörtner, 2001) and is a major consideration in our understanding of climate change impact, because the metabolic mass specific oxygen consumption rate $\left(\dot{M}O_{2}\right)$ of ectotherms generally follows that of their environment (Clarke & Johnston, 1999; Crockett & Londraville, 2006). Indeed, standard metabolic rates (SMR—the basal metabolic rate of an inactive, unfed, unstressed animal) and maximum metabolic rates (MMR—the maximal aerobic metabolic rate of an animal fully exerted) both increase across optimal temperature windows with predictable Q_10_ values (Schmidt-Nielsen, 1997; Gillooly et al., 2001; Norin & Clark, 2016). However, looking at the various components and levels of metabolism more closely reveals important details at extreme temperatures. Aerobic metabolic scope (MS—the difference between SMR and MMR) represents the energy available for non-essential, non-basal activities, such as feeding, growth, and swimming (Sandblom et al., 2014; McArley et al., 2017). MS usually becomes constrained at supra-optimal temperatures because SMR typically increases at a greater rate than MMR (Farrell, 2016). Under the oxygen- and capacity-limited thermal tolerance (OCLTT) hypothesis, the availability of an organism’s MS is the primary determinant of their thermal tolerance (Pörtner & Lannig, 2009). Whilst ectotherm metabolism increases with supra-optimal temperature, the animal’s maximum oxygen uptake ability does not, leading to hypoxemia (a lower-than-normal blood oxygen level) and a reduced MS (Pörtner & Lannig, 2009). As this reduction incurs trade-offs and constraints on aerobic-dependent activities, MS is considered a key parameter shaping the fitness of organisms subject to climate change. Additionally, at the level of individual cells, supra-optimal temperature stress increases the leakiness of mitochondrial membranes (Hardewig et al., 1999; Pörtner et al., 1999; Iftikar et al., 2015) and causes oxidative damage from the production of reactive oxygen species (Iftikar & Hickey, 2013; Fedyaeva et al., 2014). Increased membrane fluidity with supra-optimal temperatures elevates proton permeability through the inner mitochondrial membrane (Seebacher et al., 2010), thereby depressing mitochondrial membrane potential (ΔΨm) (Zukiene et al., 2010) and reducing ATP synthesis (Iftikar & Hickey, 2013). Any further increase in temperature inevitably leads to death as critical thermal optima are surpassed, with the heart being one of the first organs to fail under acute thermal stress (Iftikar & Hickey, 2013). Elevated temperatures clearly have strong metabolic impacts across various levels of biological organisation but, with increased variability and extremity of temperatures, a key consideration of any species’ resilience to climate change will be the rate at which it can attain a new steady state to compensate for environmental temperature change (i.e., the rate of thermal acclimation) (Sandblom et al., 2014). How the various rates of whole animal oxygen consumption and mitochondrial performance acclimate to thermal regime shifts thus warrants further investigation across whole animal to cellular levels of organisation.

Elevated temperatures that combine with hypoxia represent a realistic environmental condition under climate change, but there is a paucity of information on their combined effects. Increased temperature increases O_2_ demand; but the simultaneous occurrence of hypoxic conditions limits O_2_ supply, ultimately leading to a lowered rate of ATP production through oxidative phosphorylation (OXPHOS) (Devaux et al., 2019). In the absence of oxygen, some ATP can be generated via anaerobic metabolism, but this pathway is far less efficient than aerobic metabolism and can only generate ∼2 net ATP per unit of glucose compared to ∼32–36 via OXPHOS (Lehninger et al., 1993; Goldhaber, 1997; Chaudhry & Varacallo, 2018). Tissues demanding high levels of ATP, such as the heart, can only maintain anaerobic metabolism for short periods of time, while O_2_ debt and lactate will accumulate rapidly (Goolish, 1989; Gleeson, 1996). Constraints on whole animal metabolic components by temperature and hypoxia are also apparent. For example, MS is constrained at supra-maximal temperatures (outlined above) but is also suppressed by hypoxia, as MMR is directly limited by O_2_ availability (Fu et al., 2022). At extreme low O_2_ levels, the O_2_-dependent decline in MMR will eventually meet SMR (i.e., MS = 0) and the partial pressure of O_2_ (*P*O_2_) where this point of transect occurs is defined as the critical oxygen pressure (*P*_crit_) (Wu, 2002). Survival is extremely time-limited at a *P*O_2_ below *P*_crit_, as basal metabolic processes cannot be supported and the fish will rapidly reach *P*_LOE_—the *P*O_2_ at which an upright posture cannot be maintained and loss of equilibrium occurs (Mandic et al., 2013). For any study seeking to resolve the combined effects of MHW and hypoxia (e.g., hypoxia tolerance during MHW scenarios), *P*_crit_ and *P*_LOE_ will thus likely serve as useful functional indicators. Indeed, hypoxia-tolerant species are generally endowed with lower *P*_crit_ and *P*_LOE_ values (Wu, 2002; Rogers et al., 2016; McArley et al., 2019; Seibel et al., 2021) but *P*_crit_ is almost always higher at elevated temperatures, suggesting that MHW scenarios would be expected to lower hypoxia tolerance (Rogers et al., 2016).

The Australasian snapper (*Chrysophrys auratus,* or tāmure in te reo Māori) is a finfish species of high cultural, economic, and recreational importance to New Zealand (Bradford, 1999; Leach, 2006; Mossman, 2008). Indeed, the largest snapper fishery in New Zealand, SNA1 (encompassing the Hauraki Gulf, Bay of Plenty, and east coast of Northland) has a total allowable catch of 8,050 t commercially and 3,050 t recreationally (MPI, 2021). With a wide habitat distribution throughout both New Zealand and Australia, snapper are found in nearly all inshore habitats down to 200 m (Leach, 2006; Parsons et al., 2014) and, like many other sparids, encounter a broad range of natural temperatures (Francis, 1994b; Sim-Smith et al., 2013a; Parsons et al., 2014; Wakefield et al., 2015; Flikac et al., 2020; Cook et al., 2021). For example, this species has a distribution extending north to Mackay, where maximal SSTs can reach 30°C (Ferrell & Sumpton, 1993; AIMS, 2023). Temperature has important implications for the fitness, spawning, and recruitment of this species (Crossland, 1977; Scott & Pankhurst, 1992; Francis, 1993; Scott et al., 1993; Francis, 1994a; Francis, 1997; Fielder et al., 2005; Sim-Smith et al., 2013b). Snapper have been shown to be physiologically resilient to a number of stressors, including turbidity (Cumming & Herbert, 2016), high carbon dioxide (McMahon et al., 2019; 2020), and angling stress (McArley & Herbert, 2014). Whilst past research has shown snapper to be only moderately hypoxia tolerant (in terms of *P*_crit_), this species does not proactively avoid low O_2_ and will voluntarily continue to enter hypoxic water until physiological alarms are apparent (Cook et al., 2011; Cook & Herbert, 2012). Snapper are one of the widest studied fish species in New Zealand (Parsons et al., 2014) but knowledge about how they will respond to future climate change scenarios is limited. One study sought to examine the physiological impacts of a MHW on SNA1 larval snapper (McMahon et al., 2019) but, to the best of our knowledge, the 1) physiological impacts of a MHW and 2) combined effects of simultaneous hypoxia and high temperature on juvenile snapper remains unknown. With limited knowledge of thermal tolerance for this species it was assumed that snapper would struggle to cope with elevated temperatures beyond current day conditions.

This study aimed to investigate the metabolic and hypoxic tolerance of juvenile snapper from the SNA1 population by employing whole-animal (*in vivo*) and mitochondrial (*in situ*) approaches. Fish were sampled after 1 day, 10 days, and 30 days of 25°C MHW exposure and all comparisons were made against a stable 21°C control level. The study aimed to answer the following set of questions: 1) How does the metabolism of snapper change and cope in response to the thermal stress of a MHW? 2) Are the physiological reactions of snapper to MHW stress different over acute and chronic heat exposure timescales? 3) How do snapper cope when a secondary stress of acute hypoxia is applied to MHW stress? 4) Do the responses of snapper to questions (1–3) align in terms of whole-animal and mitochondrial levels of respiration? Heart mitochondria were examined because the heart of fish is usually the first organ to fail at critically high temperatures (Iftikar & Hickey, 2013) and is therefore the rate limiting organ for thermal tolerance. The overarching hypothesis was that the metabolic performance of snapper would decline when faced with MHW and hypoxic stress. For example, a reduced MS (McArley et al., 2017), increased *P*_crit_ (Schurmann & Steffensen, 1997; Collins et al., 2013), and increased mitochondrial membrane leak (Iftikar et al., 2014) were all expected after 1-day of MHW exposure, followed by partial recovery due to thermal plasticity and acclimation after 30-days chronic 25°C exposure. Understanding the response of juvenile snapper within the Hauraki Gulf of New Zealand to thermal and hypoxic stress is vital to the management of this species and the SNA1 fishery, as this area is susceptible to MHWs (Bell et al., 2023) and hypoxia (Zeldis et al., 2022).

## Materials and methods

### Animals, housing, and initial acclimation

Juvenile snapper (*C. auratus,* mean fork length (±SE) 16.9 ± 0.30 cm, body mass 114 ± 5.9 g) were caught on barbless hook and line in inshore waters (ca. 2-3 m) at Matheson’s Bay, north-eastern New Zealand, in February 2022. Following capture, fish were randomly divided between five 500 L PVC tanks with flow through filtered seawater (21°C, air saturated, 200 μm filtered, 35 ppt salinity) at the Leigh Marine Laboratory and left to acclimate (at 21°C) for just over 5 weeks. Housing was indoors with a 12 light: 12 dark artificial photoperiod. Fish were fed three times per week to satiation on crushed water-soaked commercial fish feed pellets (Ocean King 500 6.5 mm, Biomar, Tasmania, AUS). Tank water temperatures were controlled using outdoor 10–25 kW heat pumps. All experimental techniques were conducted under the approval of the University of Auckland Animal Ethics Committee (approval: 23357).

### Experimental design and protocol

At the end of the 5-week acclimation period, five experimental groups were initiated and sampled as follows: an initial 21°C control, 25°C exposure for 1 day, 25°C for 10 days, 25°C for 30 days (chronic), and a second 21°C control maintained throughout the entire experiment to day 30 (with sampling at day 30 to control for the extra time of captivity). The background (control) summertime temperature of the current study was set to 21°C, as this is the reported the present-day (2005–2011) mean February SST for the Hauraki Gulf (Evans & Atkins, 2013). The Hauraki Gulf was in a chronic MHW state for 29 weeks from 2021 to 2022, whereby SST was +2°C for ∼200 days (Bell et al., 2023). Our MHW level was selected at 25°C because the greatest SST anomaly of this 2021–2022 Hauraki Gulf MHW was +3.77°C (Bell et al., 2023) from the average summertime SST of 21°C. This indicates that the chosen level of temperature change and durations of chronic exposures are ecologically relevant. The desired temperatures over different experimental time periods were maintained with 10–25 kW heat pumps. Fish were allocated to the groups at random (12 fish per group, *n* = 60 total).

To investigate the effect of (a) high temperature acclimation and (b) hypoxia (as a secondary stressor) on snapper across a range of physiological levels, a set protocol was applied to each individual fish in the 5 treatment groups, which progressed in the following manner over a 2.5-day assessment period: 1) Transfer of individual fish to a respirometer followed by an overnight measure of standard metabolic rate (SMR) using respirometry. 2) Hypoxia challenge. 3) Overnight recovery. 4) Exhaustive chase followed by respirometry to resolve maximum metabolic rate (MMR). 5) Fast removal of fish from the respirometer, followed by euthanasia and immediate blood, tissue, and organ sampling. 6) Heart tissue mitochondrial assays. The methods involved in 1–6 are outlined in detail below.

### Thermal adjustments

Two 21°C tanks (labelled tanks 1 and 2 for the purpose of description here) were held constant at 21°C with no thermal adjustment. There were three 25°C treatment tanks (labelled tanks 3, 4, and 5) that were thermally ramped in the same way from 21°C to 25°C with an increase of 1°C per hour. Only 4 fish could be run simultaneously through the respirometry and mitochondrial assays, over a 3-day period. Thus, the start of thermal ramping for each of the three 25°C tanks was staggered in time (i.e., the increase in temperature occurred 3 days apart per tank). One-third of the fish for each of the three 25°C treatments (4 fish) were therefore sampled from each of the three 25°C holding tanks. For example, in the case of the 25°C 1 day treatment, the first 4 fish were randomly sampled from tank 3. Three days later the second 4 fish were taken from tank 4. The final 4 fish from tank 5 were sampled 3 days later. The same protocol was followed for the 25°C 10 day and 25°C 30 day treatment but the ordering from tanks 3–5 was mixed. Staggering the thermal acclimation across the tanks in such a way ensured that the allocated timeline of fish sampling after 1-, 10-, and 30-days at 25°C was exact and that fish were sampled from the 3 tanks in a randomised manner. This design was also initiated to avoid tank effects because all 3 tanks provided fish to each of the three 25°C treatments and was thus considered more robust than having each treatment attributed to only 1 tank and inexact sampling at days 1, 10 and 30. This method of staggering was also applied to the two 21°C control tanks (tanks 1 and 2) meaning that each control treatment (*n* = 12) were sampled at the appropriate time from two tanks. Again, this ensured exact sampling time and avoided tank effects because sampling was randomised between multiple tanks.

### Respirometry

Automated intermittent flow respirometry (Steffensen, 1989; Cumming & Herbert, 2016) was used to measure the mass-specific rate of O_2_ consumption $\left(\dot{M}O_{2}\right)$ of snapper in a respirometer chamber, to resolve their energetic expenditure (metabolic rate) when facing the challenge of high temperature and low O_2_. Measuring the respiratory decline of O_2_ (% air saturation) in the sealed respirometry chamber allowed the repeated measure of $\dot{M}O_{2}$ (in units of mg O_2_ kg^−1^h^−1^). Customized respirometry software (LeighResp, University of Auckland) operating on a laptop controlled the cycling of three respirometry phases: 1. Flush (flush pump turned on to clear the respirometer of waste, regain air saturation, and maintain water quality). 2. Wait (flush pump off for an initial respiratory decline in O_2_). 3. Measure (respiration now measurable according to the linear decline of O_2_ in the chamber). Phases of flush were maintained with an Eheim CompactON 1,000 pump (400–1,000 L/h). Continuous mixing within the chamber was maintained with a mixing loop consisting of an inline pump (Eheim CompactON 2,100, 1,400–2,100 L/h) connected to a short heat exchange loop of aluminium tubing that prevented the mixing pump from altering the temperature of the water within the respirometer. The % level of O_2_ saturation in the sealed respirometer was measured with a Firesting O_2_ meter (PyroScience, Aachen, Germany), connected externally though the clear chamber acrylic to an internal sensor spot in the respirometry chamber, via a bare fibre optic cable. The flush, wait, and measurement phases were cycled for approximately 4–7, 1.5, and 5 min respectively. Thus, one $\dot{M}O_{2}$ measurement was recorded every 10.5–13.5 min.

Fish were randomly selected from the given tank and their body mass (M_b_) measured in kg (with their body blotted dry but head and gills untouched). The respiration chambers were variable in size (adjusted based on M_b_), with volumes of 1.9–6.7 L (including tubing), which was on average 37.7 times the M_b_ of each individual. Fish were transferred into one of two acrylic respirometry chambers, which were contained in a larger 120 L reservoir (with 2 reservoirs and 4 chambers overall). The flow-through water into the reservoir was from the same source as that of the acclimation tanks, maintaining a constant temperature of either 21°C (mean ± SE: 20.97°C ± 0.001°C) or 25°C (25.23°C ± 0.002°C). Additional air stones within the reservoir ensured that seawater was fully air saturated. Respirometers were visually shielded from external disturbance using a shade cloth.

There was no significant difference in the mean M_b_ (ANOVA, df = 4, *F* = 0.35, *p* > 0.05), fork length (ANOVA, df = 4, *F* = 0.45, *p* > 0.05) or condition factor (ANOVA, df = 4, *F* = 0.60, *p* > 0.05) between the experimental groups. As per standard procedure, food was withheld for 48 h prior to the commencement of any respirometry, to ensure fish were starved and that $\dot{M}O_{2}$ did not contain any component of specific dynamic action (Chabot et al., 2016).

Chambers were thoroughly cleaned with ethanol between fish. Background bacterial respiration was measured in empty chambers across 3 measurement cycles (both before and after fish were added). Background respiration was assumed to increase linearly from the outset to the end of the experiment, and subtracted from each $\dot{M}O_{2}$ measurement accordingly (McArley et al., 2017).

Once fish were sealed in the chamber, respirometry measurements commenced, and fish were left alone overnight to recover from transfer stress. The SMR was resolved as the average of the lowest 10 $\dot{M}O_{2}$ values throughout the respirometry protocol (McArley et al., 2021), with most of these values usually recorded during the overnight periods. The $\dot{M}O_{2}$ (mg O_2_ kg^−1^ h^−1^) was calculated using the linear slope of the O_2_ decline within the respirometer during the closed measurement period, by the following equation:

$$\dot{M}O_{2}=\frac{\left(V_{r}-V_{f}\right)\times \frac{\Delta \%Sat}{t}\times \alpha }{M_{b}}$$

Where V_r_ represents the respirometry chamber volume and V_f_ the volume of the fish (l, assuming that 1 g of fish is equivalent to 1 mL of water), Δ%Sat/t is the change in O_2_ (% air saturation) per unit time h), α is the solubility coefficient of O_2_ in water (mg O_2_% O_2sat_^–1^ 1^–1^; salinity 35 ppt, either 21 or 25°C) and M_b_ is the fish mass (kg) (Schurmann & Steffensen, 1997; Devaux et al., 2019). The SMR Q_10_ (the rate that SMR changes with every 10 °C temperature change) was calculated as:

$$Q_{10}={\left({SMR}_{1}-{SMR}_{2}\right)}^{2.5}$$

Whereby the difference between the SMR values at the two temperatures is raised to the power of 2.5 (as $\frac{10}{\Delta temperature}$ ) (Laspoumaderes et al., 2022).

During respirometry, O_2_ changes were measured in % air saturation. Such values were then converted into O_2_ pressure units (*P*O_2_ in kPa) based on the measured atmospheric pressure on the day.

### Hypoxia challenge

The hypoxia challenge was given to the fish on the second day of respirometry, whereby the LeighResp software was manually overridden and held in a continuous closed ‘measurement’ phase (i.e., flush pump off), which allowed the fish to gradually draw down the chamber’s O_2_ through respiration for 38–57 min, with the fish under constant supervision. As O_2_ declined and hypoxia progressed, the fish eventually shifted from a state of oxyregulation (where SMR could be maintained steady) to a state of oxyconformation (where SMR was forced to reduce with the severely low levels of O_2_ in the surrounding seawater) (Farrell & Richards, 2009). The point of inflection where stable resting $\dot{M}O_{2}$ values could no longer be maintained and fish transitioned from a state of oxyregulation to oxyconformation was identified and recorded as the critical O_2_ pressure (*P*_crit_) (Farrell & Richards, 2009). To find the *P*_crit_ of each fish, $\dot{M}O_{2}$ values below SMR were plotted and a linear regression was performed on the data. The % air saturation level (then converted to *P*O_2_, as detailed above) where the linear regression intercepted with SMR was taken as the *P*_crit_ for that fish. The hypoxia challenge was continued beyond *P*_crit_ with O_2_ declining until the fish experienced loss of equilibrium (LOE) for 10 s. The *P*O_2_ where fish showed LOE was recorded as *P*_LOE_. Once *P*_LOE_ was identified, the flush pump was turned on and the chamber was replenished with aerated seawater until full oxygenation was regained. Respirometry was then switched back to the original automated intermittent flush-wait-measurement cycles, and the fish was left to recover overnight. $\dot{M}O_{2}$ values during this recovery period were then taken for comparative measures of anaerobic tolerance and recovery. Four parameters of O_2_ debt were calculated from the fish recovering in the respirometers overnight: 1) Peak $\dot{M}O_{2}$ when recovering from *P*_LOE._ 2) Total O_2_ debt. 3) The time taken to recover to SMR. 4) The total O_2_ debt recovered per hour. Absolute SMR was not used as the point of recovery but was rather assumed to be SMR +10% to account for the inevitable increase in movement of the snapper as they were recovering (i.e., snapper rarely returned to SMR, but instead stayed a little above SMR because they engaged in some spontaneous activity in the chambers). The total O_2_ debt was calculated as the total area under the $\dot{M}O_{2}$ recovery curve. Graphpad Prism was used to integrate all the areas sandwiched between each consecutive $\dot{M}O_{2}$ measure and SMR +10%, and the areas between each two consecutive points were summed. The total O_2_ debt recovered per hour was calculated by dividing the total O_2_ debt by the time taken to return to SMR.

### Exhaustive exercise

Once fish had recovered from the hypoxia challenge their MMR was determined using an exhaustive exercise protocol (Khan et al., 2014; McArley et al., 2018). Each fish was chased to exhaustion with light tail touches for either 5 minutes or until it no longer performed burst swimming to escape the touch. After resealing the chamber, measurement phases were cycled manually over a timeframe of ∼10 min to ensure that the saturation of O_2_ in the chamber did not drop below 80% in any of the measurement cycles. Once MMR was ascertained, each fish was left to recover for 2-3 h before the whole respirometry protocol was terminated. MMR was taken as the highest $\dot{M}O_{2}$ value recorded for each fish across the whole respirometry protocol and was almost always the first $\dot{M}O_{2}$ value after exhaustive exercise. MS was calculated as the difference between SMR and MMR.

### Blood and tissue sampling and analysis

Following completion of the respirometry protocol, fish were removed from the respirometers and euthanized via “ike jimi” (Wells & Weber, 1990). Fish were placed immediately on ice.

Blood was sampled immediately following death via caudal venepuncture with a 2.5 mL heparinised syringe fitted with a 21 gauge hypodermic needle (as per McArley and Herbert (2014)). Blood was then transferred to a 2 mL Eppendorf tube, sealed, and immediately placed on ice for subsequent analysis and processing. The heart ventricle (atrium removed), spleen, and liver were removed and weighed (wet weight).

Haematocrit (Hct) was measured by spinning a 75 mm capillary tube full of whole blood in a haemofuge (Haemocentaur, MSE, UK) for 3 min (12000 g) and measuring the compacted percentage of red blood cells within the tube with a measurement rule. Blood haemoglobin concentration ([Hb]) was measured spectrophotometrically using modified Drabkins reagent, whereby a 10 μL whole blood subsample was mixed with 1 mL of Drabkins and absorbance was measured at 540 nm after 10 min (Wells & Dunphy, 2009).

The relative organ mass (%) of the spleen, liver and ventricle, and the fish condition factor, were calculated as:

$$Relative\;organ\;mass=\frac{M_{organ}}{M_{b}}\times 100$$

$$Condition\;factor=\left(\frac{M_{b}}{{FL}^{3}}\right)\times 100$$

Where M_organ_ is the wet mass of either the liver (kg), spleen or heart ventricle, M_b_ is the fish body mass (kg), and FL is the fork length m).

Mean corpuscular haemoglobin (MCH, g L^−1^) was calculated as:

$$MCH=\frac{Hb}{HCT}\times 100$$

Where Hb is the haemoglobin (g L^−1^) and HCT is the haematocrit (%).

### Mitochondrial respirometry

Following dissection, the heart was placed immediately in ice-cold, modified (to account for the higher osmolarity of marine teleosts compared to mammals) respiratory medium MiR05, with a final composition of 0.5 mM EGTA, 60 mM lactobionic acid, 20 mM taurine, 10 mM KH2PO4, 20 mM HEPES, 160 mM sucrose, 1 g/L BSA, pH 7.24 at 20°C (Gnaiger et al., 2000; Iftikar et al., 2010). The heart tissue was quickly blotted dry and weighed prior to being homogenized in cold MiR05 using a motorised homogeniser. Mitochondrial respiration assays were then run with a tissue concentration of 5 mg/L heart homogenate, within the 2 mL Oroboros Oxygraph-2k (O2k) thermostated respirometer chamber. Assay temperature was maintained throughout the experiment at the fish’s acclimation temperature (either 21°C or 25°C). Oxygen calibration was performed at 100% air saturation (101.1 kPa) according to the chamber temperature at the outset of each assay. Data was recorded using Datlab 7.1 software (Oroboros instruments, Innsbruck, Austria). Mass-specific mitochondrial respiration flux (JO_2_, pmol/(s.mg)) was calculated in real time by DatLab, as the negative time derivative of the O_2_ concentration.

### Assay protocol

**FIGURE 1:**
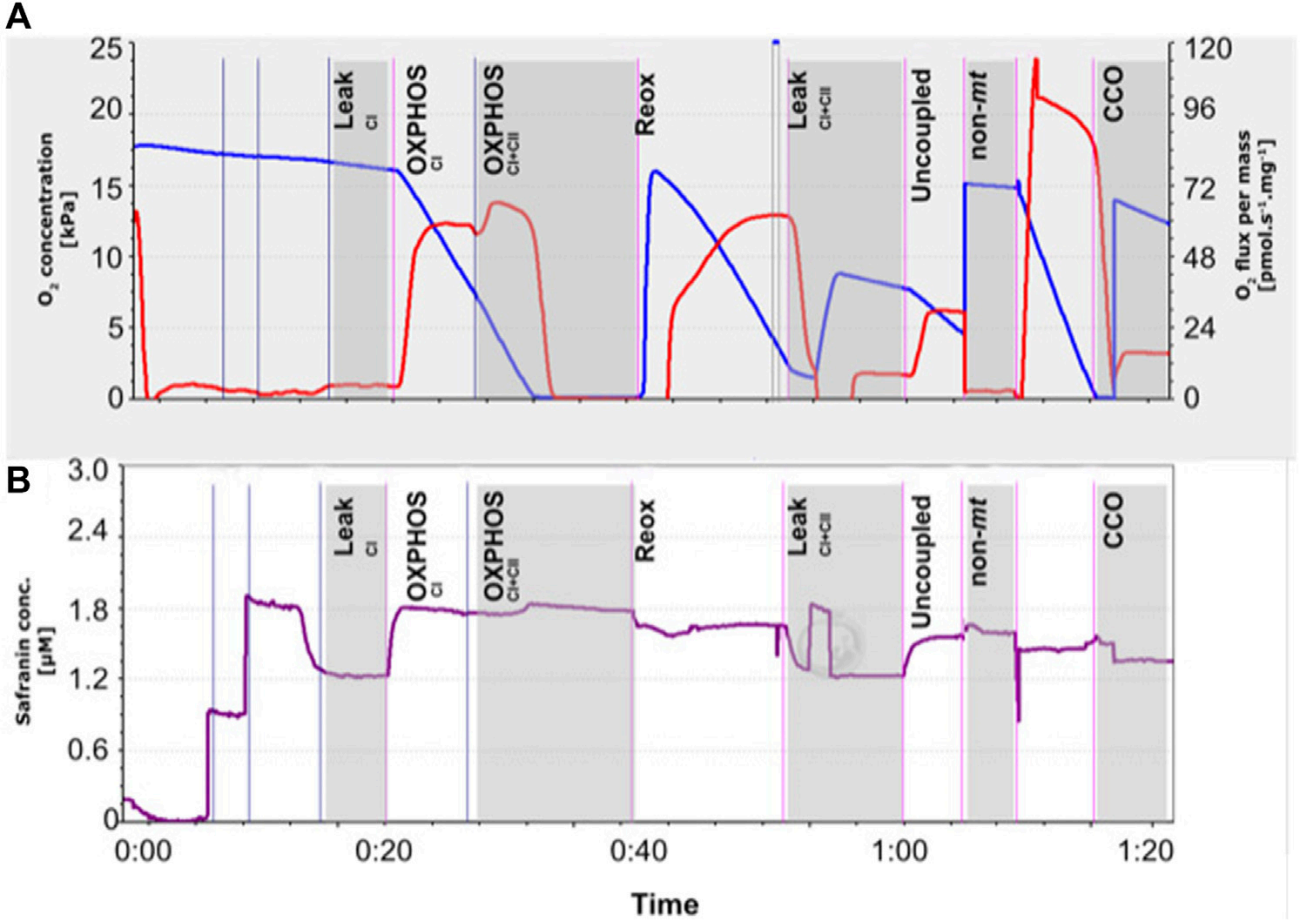
Representative trace of the mitochondrial respirometry assay performed on permeabilized snapper heart tissue (5 mg), conducted using Oroboros™ O2k respirometers, as shown in real-time by the Datlab 7.1 software. **(A)** Chamber oxygen concentration (blue line) and its derivative, i.e., oxygen consumption (JO_2_, red line) corrected for mass of the sample. **(B)** Mitochondrial membrane potential (ΔΨm) was monitored in alongside JO_2_ using calibrated-safranin concentration (up to 2 μM) fluorescence. Safranin accumulates within mitochondria as ΔΨm increases, which mediates a decrease in fluorescence. Sequential titration of substrates, inhibitors and uncoupler were used to induce mitochondria at various states specified in vertical black labels. The Leak_CI_ (complex I) state was induced by the addition of saturating concentrations of pyruvate and malate, before ADP activated CI OXPHOS, and succinate allowed OXPHOS from both CI and CII. The chambers were allowed to run into anoxia (for a short period) to calculate the *P*50_mito_, before reoxygenation with H_2_O_2_ and catalyse. To test for mitochondrial coupling, oligomycin was used to induce the Leak_CI+CII_ state, followed by carbonyl cyanide m-chloro phenyl hydrazone (CCCP) to determine the maximum capacity of the electron transport system. Background oxygen consumption was then measured following antimycin A addition. Ascorbate (Asc) and *N*,*N*,*N*′,*N*′-tetramethyl-*p*-phenylenediamine (TMPD) were then added to measure maximum cytochrome c oxidase (CCO) rates, with sodium azide (AZ) thereafter to inhibit CCO and account for background TMPD auto-oxidation rates.

The assay protocol is visually outlined in Figure 1, whereby the mitochondrial JO_2_ and chamber oxygen concentration (Figure 1A) and the change in cationic fluorophore safranine-o (safr, Amp uM, Figure 1B) at each step of the assay (detailed below) were displayed in real-time by the DatLab software.

Following sample addition and safranin (up to 2 μM) fluorescence calibration, assay protocol steps (Figure 1) were performed as follow: 1) Pyruvate and malate were added (respectively 5 and 2 mM) to measure complex I (CI) leak state. 2) The addition of adenosine diphosphate (ADP, 2.5 mM) engaged ATP synthase, thus allowing CI OXPHOS measurement. 3) Succinate (10 mM) began complex II (CII). With CI already running in the background, this step therefore measured CI + CII OXPHOS together. Mitochondria were then allowed to draw the chamber O_2_ down through respiration until anoxia, so that the mitochondrial *P*50 (*P*50_mito_, the *P*O_2_ where mitochondrial O_2_ consumption is at 50% of maximal rates (Chung et al., 2017)) could be calculated. To avoid any potential mitochondrial damage due to anoxia, the anoxic period did not exceed 10 min, as per previous research by Lau et al. (2017). 4) To reoxygenate without interference to the fluorescent signal, H_2_O_2_ (5 μL, 0.75%) and catalase (5 U/ml) were added. Throughout the rest of the assay, oxygenation was kept above ∼80% air saturation (i.e., >160 mM and >190 mM for 21ºC and 25°C respectively). 5) Oligomycin (10 nM) then inhibited ATP synthase to allow the measurement of proton leak with CI and CII substrates (Leak_CI+CII_). 6) Carbonyl cyanide m-chloro phenyl hydrazone (CCCP, 0.1 µM) was added to measure the ‘uncoupled’ respiration state. CCCP is a lipophilic weak acid and acts to exchange protons across the inner mitochondrial membrane. It therefore dissipates proton gradients, bypassing proton flow through the ATP synthase and decreasing the membrane potential. 7) Antimycin A (2.5 µM) was then added to block the electron pathway at complex III and allow for the measurement of background oxygen consumption. 8) Ascorbate (Asc) and *N*,*N*,*N*′,*N*′-tetramethyl-*p*-phenylenediamine (TMPD) were then added (2 and 0.5 mM, respectively) measure maximal cytochrome *c* oxidase (CCO) rates. 9) Sodium azide (AZ, 100 mM) thereafter inhibited CCO to measure background TMPD auto-oxidation rates. CCO net rates were determined as (TMPD + Asc) – (AZ).

A 2-min average for JO_2_ and ΔΨm were sampled at each step of the assay and extracted from DatLab, for further analysis in Excel. Non-mitochondrial respiration determined at 7) was subtracted from the flux at each state, but did not differ between treatment groups (ANOVA, df = 4, *F* = 0.73, *p* > 0.05). The respiratory control ratio (RCR), as a proxy for mitochondrial efficiency, was calculated according to Gnaiger (2012):

$$RCR=\frac{CI+CII\;OXPHOS}{CI+CII\;Leak}$$

To determine the *P*50_mito_, a section of the *P*O_2_ and respiration flux data was extracted following attainment of maximal flux after succinate addition, and then few minutes of complete anoxia to determine the respiration profiles as *P*O_2_ declined. Hill-curve fitting was performed in Python environment with the fitted respiration curves used to find the *P*50_mito_ (code availability: https://github.com/julio0029/Snapper_Study_2022_TM, using pandas, scipy and pingouin packages). The hill curve equation fitted well to these data (93.3% of values had an R^2^ >70%) and only hill curves with an R^2^ greater than 70% were used (mean R^2^ = 97%).

### Mitochondrial membrane potential

The mitochondrial membrane potential (ΔΨm, mV) was estimated simultaneously with JO_2_ throughout the assay using safr fluorescence, as per Devaux et al. (2019). As a cationic dye which is permeable to mitochondrial membranes, safr has a fluorescent quench during its movement through the inner-mitochondrial membrane, from the intermembrane space to the mitochondrial matrix (Åkerman & Wikström, 1976; Zanotti & Azzone, 1980). Estimates of ΔΨm can be derived from a near-linear correlation between spectral shift of safr and the energized state of the mitochondria, whereby an increase in safr concentration within the mitochondria due to increase in ΔΨm mediates a decrease in fluorescence (Devaux et al., 2019). Calculations of ΔΨm were conducted using the Nernst equation by the methods previously described by Devaux et al. (2019) and Pham et al. (2014), whereby:

$$\Delta \Psi m=2.3026\;x\;\left(\frac{RT}{zF}\right)\;x\;\mathrm{Log}10\left(safr\;out\;/\;safr\;in\right)$$

Where R is the gas constant (8.314 J.mol^−1^.K^−1^), T is the temperature (in Kelvin), z is the valence state of the ion (+1) and F is the Faraday constant (96,485.340 C.mol^−1^). “Safr out” (μM) is the safranin concentration outside of the mitochondria. The concentration of safranin inside the mitochondrial matrix, “safr in” (µM) is dependent on the volume of the mitochondria. Mitochondria are known to constitute 19.8% ± 0.8% of snapper heart tissue (Cook et al., 2013); thus, ca. 20% of mitochondrial tissue volume was used in the heart calculations. The work undertaken to maintain mitochondrial respiration was calculated as JO_2_/-ΔΨm (pmol O_2_/(s*mg*mV), as per Devaux et al. (2019).

### Statistical analyses

T-tests were initally run on each variable between the 21°C control from the outset and end of the experiment, to account for any effect of time in captivity. Only the relative liver mass returned a signficiant difference between the two controls, thus the two controls were analysed separately for this parameter. All other parameters returned no significant difference between the two controls (*p* > 0.05) and were thus combined (pooled) for further statistical analyses. Outliers (<2 standard deviations away from mean) were removed (Norin et al., 2014; Oellermann et al., 2020). Data was normality checked with a Shapiro-Wilk and homogeneity of variances with a Bartlett’s test. Unless otherwise stated, one-way ANOVAs were then used to test the effect of increased temperature on every parameter and a Tukey’s multiple comparisons test was run to detect individual *post hoc* differences. Where normality was confirmed but the variances were significantly different (violating the assumptions of ANOVA), Welch (1951) ANOVA tests were instead run, with a *post hoc* Games-Howell’s multiple comparison test where necessary. In the rare instance where normality was not met (including when data was log transformed) then a non-parametric Kruskal-Wallis test was performed, with a Dunn’s *post hoc* multiple comparisons test. All statistics were run through GraphPad Prism v8.0.2 and statistical significance was accepted at *p* < 0.05.

## Results

### Whole animal respirometry

After raising temperature from 21°C to 25°C, SMR, MMR, and MS were all increased significantly (each *p* > 0.01; Table 1)—with an average increase of 30% for SMR, 20% for MMR, and 16% for MS—with no additional significant differences between any of the 25°C groups (*p* < 0.05). All SMR Q_10_ values were within the range of 1.74–2.11 (Table 1).

**TABLE 1:** Standard metabolic rate (SMR), maximum metabolic rate (MMR), aerobic metabolic scope (MS), and SMR Q_10_ of snapper, from the 21°C control to 25°C exposure for 1-, 10-, and 30-days. Data presented as mean ± SE of 11-12 individuals per experimental group and 24 individuals per control (beginning and end control groups combined). Significant differences (*p* < 0.05, main effects test details in bottom row) are indicated by different superscript letters.

Treatment	SMR (mg O_2_ kg^−1^ h^−1^)	MMR (mg O_2_ kg^−1^ h^−1^)	MS (mg O_2_ kg^−1^ h^−1^)	SMR Q_10_
21°C Control	132.3 ± 4.3^a^	464.2 ± 7.5^a^	331.9 ± 7.5^a^	—
25°C 1-Day	161.4 ± 4.3^b^	538.6 ± 11.7^b^	373.4 ± 9.7^b^	1.74
25°C 10-Days	178.3 ± 10.0^b^	557.4 ± 16.1^b^	379.0 ± 8.9^b^	2.11
25°C 30-Days	175.3 ± 5.0^b^	579.0 ± 8.9^b^	403.7 ± 9.5^b^	2.02
Main Effects Test	Welch’s ANOVA, df = 3, *W* = 16.20, *p* < 0.01	ANOVA, df = 3, *F* = 27.29, *p* < 0.01	ANOVA, df = 3, *F* = 13.44, *p* < 0.01	—

### Hypoxia tolerance

**FIGURE 2:**
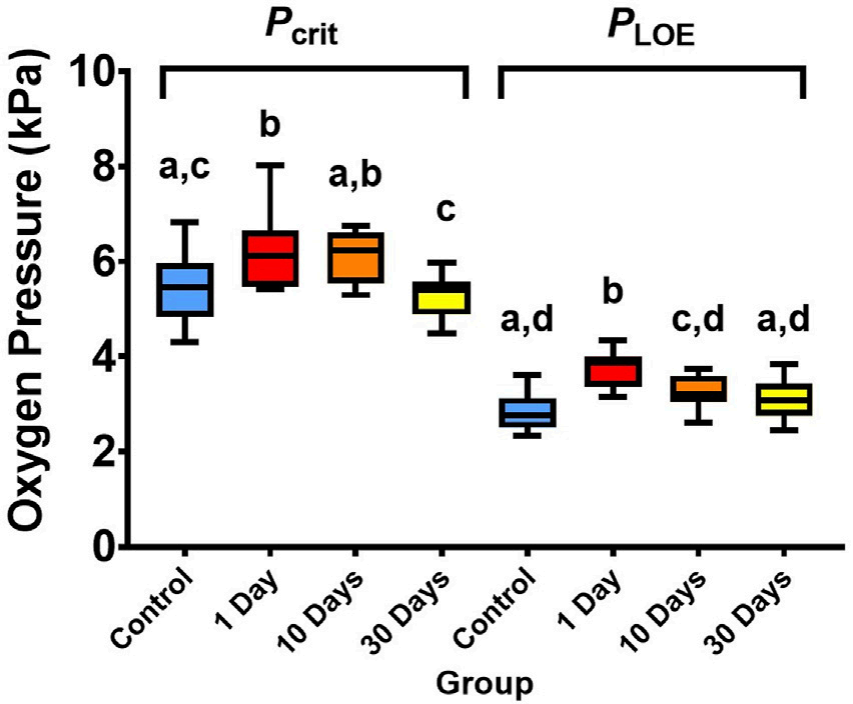
Critical oxygen tension (*P*_crit_) and partial pressure of environmental oxygen where fish loses equilibrium (*P*_LOE_) of snapper at 21°C and 25°C after 1-, 10, and 30-days of acclimation exposure. Data presented as 75th, 50th (median), and 25th percentiles ± minimum and maximum of 11-12 individuals per treatment group and 23-24 individuals per control (beginning and end control groups combined). Significant differences (*p* < 0.05, tested with one-way ANOVA) are indicated by different superscript letters.

*P*_crit_ varied significantly between the temperature treatments (ANOVA, df = 3, *F* = 6.48, *p* < 0.01; Figure 2), whereby it was 14% higher (i.e., showed signs of decreased hypoxia tolerance) when temperature was increased from 21°C to 25°C for only 1 day (*p* < 0.01). However, *P*_crit_ recovered to control levels with 10 and 30 days at 25°C (*p* > 0.05). *P*_LOE_ also diffed between the temperature treatments (ANOVA, df = 3, *F* = 17.57, *p* < 0.01; Figure 2), with higher values (showing less hypoxia tolerance) following 1- and 10-days of exposure to warmer 25°C temperatures (each *p* < 0.01; 33% and 16% higher than the control following 1- and 10-days respectively). However, after 30-days of 25°C exposure, *P*_LOE_ also recovered with no significant difference from the control (*p* > 0.05).

**FIGURE 3:**
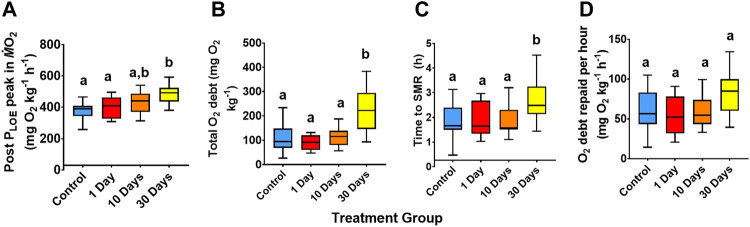
Recovery variables following *P*_LOE_ for snapper at 21°C and 25°C for 1-, 10, and 30-days acclimation exposure. **(A)** Post *P*_LOE_ peak in $\dot{M}O_{2}$ , **(B)** total oxygen debt, **(C)** time taken to return to standard metabolic rate (SMR), and **(D)** the calculated oxygen debt repaid per hour. Data presented as 75th, 50th (median), and 25th percentiles ± minimum and maximum of 11-12 individuals per treatment group and 22–24 individuals per control (beginning and end control groups combined). Significant differences (*p* < 0.05, tested with ANOVA or, in the case of total O_2_ debt, Welch’s ANOVA) are indicated by different superscript letters.

In terms of *P*_LOE_ recovery (Figure 3), significant differences occurred between treatments, whereby the 30-day 25°C treatment was greater than the other groups, in terms of the post *P*_LOE_ *Ṁ*O_2_ peak (29.5% greater than the control; ANOVA, df = 3, *F* = 9.96, *p* < 0.01; Figure 3A), total oxygen debt (105% greater than the control; Welch’s ANOVA, df = 3, *W* = 6.42, *p* < 0.01; Figure 3B), and the time taken to recover $\dot{M}O_{2}$ to SMR (46% greater than the control; ANOVA, df = 3, *F* = 4.07, *p* = 0.01; Figure 3C). The longer time to recover to SMR for the 30-day 25°C treatment was likely due to the greater total oxygen debt, especially as the quantity of oxygen debt repaid per hour was the same across groups (ANOVA, df = 3, *F* = 2.42, *p* > 0.05; Figure 3D).

### Blood and organ indices

The main effects test for Hct revealed a significant difference between the groups, but the only specific difference was a 13% increase in the percentage of Hct after 1-day of warming to 25°C compared to the control (*p* = 0.03; Table 2). [Hb], Hct, and relative spleen mass did not vary (Table 2). Relative ventricular mass was significantly different between the temperature treatments (Table 2), with the control fish at 21°C having a 14% smaller heart on average than the 10- (*p* = 0.02) and 30-days (*p* < 0.01) 25°C exposure treatments. In terms of relative liver mass, the second 21°C control group was far smaller than that of the first control (*t* = 2.46, df = 22, *p* = 0.02; Table 2), thus the two controls were presented separately. The main effects test on the acclimation groups detected a significant difference across all groups in liver mass, whereby the initial 21°C control had a significantly larger relative liver mass than both the 30-days 25°C treatment (81% larger; *p* < 0.01; Table 2) and the second 21°C control (62% larger; *p* = 0.01; Table 2).

**TABLE 2:** Blood and organ indices for snapper acclimated at 21°C and 25°C for 1-, 10, and 30-days. The Hct (haematocrit), [Hb] (haemoglobin), MCH (mean corpuscular haemoglobin), relative ventricular mass, relative liver mass, and relative spleen mass. Data presented as mean ± SE of 10–12 individuals per experimental group and 19–24 per control (beginning and end controls combined), except for the liver, whereby the two controls (11-12 individuals each) remained separate due to statistical difference. Significant differences (*p* < 0.05, main effects test details in bottom row) are indicated by different superscript letters.

Treatment	Hct (%)	[Hb] (gL^−1^)	MCH (gL^−1^)	Relative spleen Mass (%)	Relative ventricular Mass (%)	Relative liver Mass (%)
21°C Controls (pooled)	28.7 ± 0.7^a^	73.2 ± 1.4^a^	251.8 ± 2.5^a^	0.04 ± 0.002^a^	0.060 ± 0.002^a^	1.9 ± 0.2^a^ (1st control)
25°C 1-Day	32.3 ± 1.0^b^	77.5 ± 1.1^a^	243.7 ± 4.8^a^	0.04 ± 0.003^a^	0.065 ± 0.001^a,b^	1.5 ± 0.7^a,b^
25°C 10-Days	29.6 ± 0.6^a,b^	76.7 ± 2.0^a^	252.4 ± 8.3^a^	0.04 ± 0.003^a^	0.068 ± 0.002^b^	1.3 ± 0.1^a,b^
25°C 30-Days	30.3 ± 0.8^a,b^	76.2 ± 2.1^a^	251.8 ± 3.5^a^	0.04 ± 0.001^a^	0.071 ± 0.003^b^	1.1 ± 0.07^b^
21°C 2nd Control	—	—	—	—	—	1.2 ± 0.10^b^
Main Effects Test	ANOVA, df = 3, *F* = 3.8, *p* = 0.02	ANOVA, df = 3, *F* = 1.4, *p* > 0.05	Welch’s ANOVA, df = 3, *W* = 0.78, *p* > 0.05	ANOVA, df = 3, *F* = 1.7, *p* > 0.05	ANOVA, df = 3, *F* = 6.8, *p* < 0.01	Kruskal-Wallis ANOVA, df = 4, *H* = 19.79, *p* < 0.01

### Mitochondrial respiration

The O_2_ flux (JO_2_) of heart mitochondria did not statistically differ between treatments for either CI or CI + CII OXPHOS (Table 3). The temperature increase from 21°C to 25°C did not incur additional significant CI and CII + CII OXPHOS ΔΨm variation; thus, there was no change to the energetic work required by the mitochondria to maintain the membrane potential (Table 3). The second control could not be included in the analyses for ΔΨm or work, due to systematic error, thus only the first control was analysed for these data.

**TABLE 3:** Heart mitochondrial oxygen consumption rates (JO_2_), mitochondrial membrane potential (ΔΨm) and translated work (JO_2_/ΔΨm) of snapper acclimated to marine heat wave scenario treatments. Oxidative phosphorylation (OXPHOS) was achieved with either electron inputs at complex I (CI OXPHOS; with saturated pyruvate, malate and ADP) and with additional complex II input (CI + CII OXPHOS; added succinate). Data presented as mean of 8 individuals per group ±SE. Significant differences (*p* < 0.05, main effects test details in bottom row) are indicated by different superscript letters.

	CI OXPHOS			CI + CII OXPHOS		
Treatment	JO_2_ (pmol/(s*mg))	ΔΨm (mV)	Work (pmol O_2_/(s*mg*mV)	JO_2_ (pmol/(s*mg))	ΔΨm (mV)	Work (pmol O_2_/(s*mg*mV)
21°C Control 1	53.3 ± 2.5^a^	−147.5 ± 2.1^a^	0.4 ± 0.05^a^	66.3 ± 3.1^a^	−145.6 ± 7.9^a^	0.4 ± 0.06^a^
25°C 1-Day	50.2 ± 3.8^a^	−144.9 ± 3.4^a^	0.4 ± 0.03^a^	48.4 ± 6.5^a^	−146.5 ± 3.0^a^	0.3 ± 0.06^a^
25°C 10-Days	55.6 ± 6.1^a^	−139.1 ± 7.8^a^	0.4 ± 0.06^a^	68.3 ± 6.2^a^	−137.2 ± 8.6^a^	0.5 ± 0.07^a^
25°C 30-Days	60.3 ± 8.8^a^	−135.0 ± 6.5^a^	0.5 ± 0.05^a^	56.1 ± 7.0^a^	−136.0 ± 6.7^a^	0.5 ± 0.05^a^
Main Effects Test	Welch’s ANOVA, df = 3, *W* = 0.44, *p* > 0.05	Kruskal-Wallis ANOVA, df = 3, *H* = 2.26, *p* > 0.05	ANOVA, df = 3, *F* = 1.43, *p* > 0.05	ANOVA, df = 3, *F* = 2, *p* = 0.06	Kruskal-Wallis ANOVA, df = 3, *H* = 0.73, *p* > 0.05	ANOVA, df = 3, *F* = 1.10, *p* > 0.05

**FIGURE 4:**
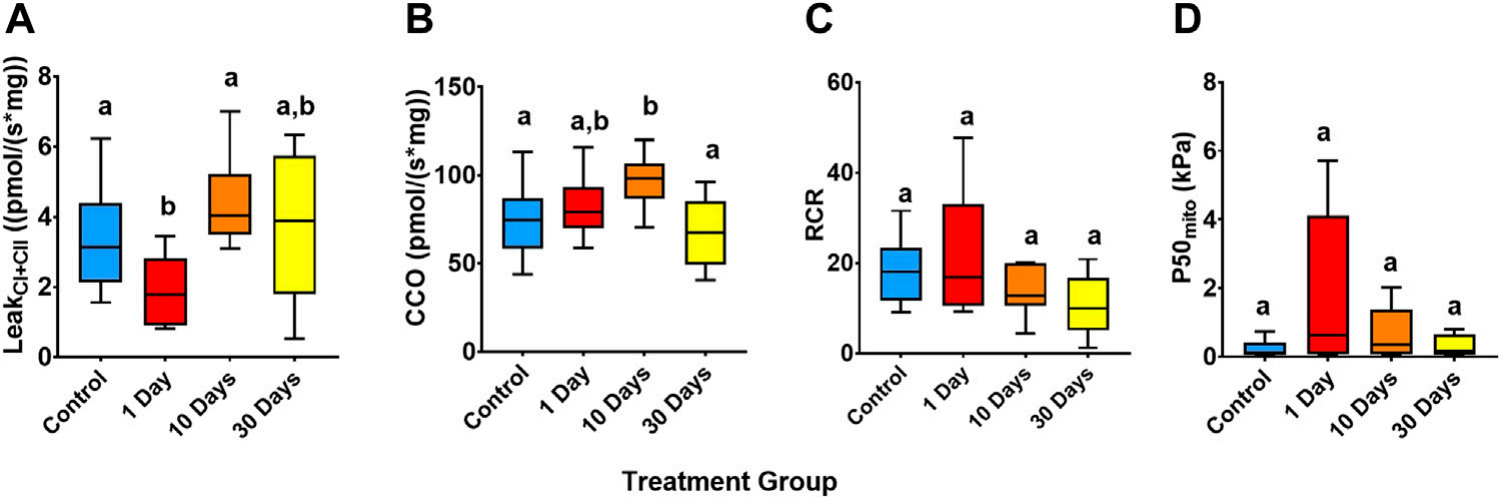
Snapper heart mitochondrial performance of snapper acclimated to 21°C and at 25°C for 1-, 10, and 30-days. **(A)** O_2_ flux mediated by mitochondrial complex I and complex II inputs and not directed to oxidative phosphorylation (Leak_CI+CII_). **(B)** Absolute cytochrome c oxidase (CCO) rates. **(C)** The respiratory control ratio (RCR, measured as CI + CII OXPHOS/Leak_CI+CII_). **(D)** Mitochondrial *P*50 (*P*50_mito_), as the partial pressure of O_2_ where mitochondrial O_2_ consumption is at 50% of maximal rates. Data presented as 75th, 50th (median), and 25th percentiles ± minimum and maximum of 8–11 individuals per experimental group and 11–18 individuals per control (beginning and end controls combined). Significant differences (*p* > 0.05, tested by ANOVA (CCO), Welch’s ANOVA (Leak_CI+CII_ and RCR), and Kruskal-Wallis test (*P*50_mito_)) are indicated by different superscript letters.

Leak_CI+CII_ differed significantly between treatments (Welch’s ANOVA, df = 3, *W* = 8.90, *p* = 0.01; Figure 4A). Indeed, Leak_CI+CII_ declined (became more efficient) by 44% from the control following 1-day of 25°C (*p* = 0.03), but the control level was restored by 10-days at 25°C (*p* > 0.05). The CCO flux also differed between the treatments (ANOVA, df = 3, *F* = 6.04, *p* < 0.01; Figure 4B), with the control 24% lower than the 10-day 25°C group (*p* < 0.01), indicating a greater OXPHOS output from complex IV in this group.

The respiratory control ratio (RCR) for snapper heart mitochondria was high throughout the experiment, ranging from 10.6 to 21.6 (indicating tight and efficient coupling of respiration to ATP synthesis), with no significant difference between groups (Welch’s ANOVA, df = 3, *W* = 3.13, *p* > 0.05; Figure 4C). Moreover, *P*50_mito_ (Kruskal-Wallis ANOVA, df = 3, *H* = 3, *p* > 0.05; Figure 4D) did not return any significant difference between treatments.

## Discussion

Marine heat waves (MHWs) are expected to increase in frequency and severity as climate change advances, with anticipated impact on marine ecosystems (Oliver et al., 2018). This study sought to investigate the metabolic response of SNA1 stock snapper to heat shock at the organism and mitochondrial levels. Additionally, we tested whether progressive acclimation would allow a greater degree of thermo- and hypoxia-tolerance for this species. The findings of this study were unexpected as exposure to 25°C, a realistic MHW SST scenario for the Hauraki Gulf of New Zealand, did not constrain the metabolic O_2_ uptake rate of snapper, nor change their condition factor. Indeed, 25°C may actually be more within the optimal temperature range of this species—which has a global range that extends from warmer sub-tropical to cold temperate waters—than the present-day (February) average of 21°C. Our findings in part contrast with the initial hypothesis that snapper performance would rapidly decline at the beginning of a 25°C MHW and then undergo partial recovery following 30-days of high temperature exposure. In keeping with reports from previous workers, we made the assumption that an expanded aerobic metabolic scope (MS) benefits fish fitness performance. Here we report a 16% increase in MS, on average, as temperature increased from 21°C to 25°C. Moreover, MS increased 1-day into the MHW and remained high over the 30 days of the challenge. Regardless of acclimation period, fish in the 25°C treatment showed elevated SMR and MMR relative to 21°C controls. Moreover, the fold increase in MMR capacity exceeded the relative increase in SMR and this resulted in expansion of MS at 25°C. The average SMR Q_10_ of 1.96 from 21°C to 25°C was within the range of passive, direct effects of temperature (Clarke & Johnston, 1999; Schulte, 2015; Reeve et al., 2022), supporting assumptions that snapper are capable to acclimate. Using whole animal O_2_ uptake rates as a performance predictor, north-eastern New Zealand snapper stocks may even metabolically benefit from climate change (Watson, 2018).

The strong metabolic resilience of snapper is also reinforced by the maintenance of heart mitochondrial performance throughout all stages of the MHW exposure. Regardless of substrate input, OXPHOS was unchanged during the warm acclimation. However, mitochondrial respiration not attributed to OXPHOS (i.e., Leak_CI+CII_) decreased at 1-day 25°C acclimation, suggesting a greater utilisation of O_2_ flux toward ATP production (Gnaiger, 2012). CCO rates were greater in the 10-day 25°C treatment than in the control, suggesting a potential upregulation of cytochrome c oxidase output, which did not translate to an increase in oxidative phosphorylation. CCO rates however returned similar to that of 21°C acclimated fish after 30-days acclimation at 25°C. Regardless of acclimation, CCO rates were ∼2-3 fold greater than OXPHOS rates. This CCO-excess capacity in the heart means that the transitional increase in CCO rates had insignificant effect on mitochondrial respiration (Gnaiger et al., 1998). Regardless, all states recovered to be no different from the 21°C control after 10- and 30-days at 25°C. Heart mitochondria also showed no change between groups in *P*50_mito_, RCR, or workload in terms of JO_2_ per ΔΨm (Table 3), suggesting that mitochondria retained a certain efficiency towards ATP production.

A common limitation of using $\dot{M}O_{2}$ as a proxy for ATP generation is the assumption that oxygen uptake is equivalent to coupled ATP production (Brand et al., 1994). It is well known that mitochondrial membranes appear leakier at higher temperatures, resulting in a disconnect between the amount of ATP produced and O_2_ consumed with rising temperatures (Iftikar & Hickey, 2013; Salin et al., 2015). Whilst largely ignored, when seeking to draw solid conclusions from thermal challenge $\dot{M}O_{2}$ data, it is important to ensure that additional proton leak does not occur in the inner mitochondrial membrane. By measuring the change in membrane potential at each state and, therefore, calculating the mitochondrial work undertaken through cellular respiration at both the whole-animal and mitochondrial levels, this research exceeds the usual limitations of respirometry by demonstrating that the metabolic resilience of snapper to MHWs is consistent across multiple physiological levels. As the heart is usually the first organ to fail from supra-optimal temperatures (Iftikar & Hickey, 2013), the sustained performance of the snapper heart clearly indicates that snapper can tolerate 25°C for at least 30 days.

As the frequency of aquatic hypoxia events are projected to increase into the future (Keeling et al., 2009), a species’ physiological plasticity for hypoxia tolerance will be a key determinant of performance (Rogers et al., 2016), and we hypothesised that thermal challenge would impact physiological responses (e.g., Schurmann & Steffensen, 1997). While there were elevations in *P*_crit_ and *P*_LOE_ following acute hypoxic challenge, these increases were relatively small (+0.78 and +0.94 kPa from the control for *P*_crit_ and *P*_LOE_ respectively). Overall, snapper appeared to recover and maintain hypoxia tolerance at 25°C exposures. The 30-day 25°C temperature treatment group appeared to build up a greater total oxygen debt and a greater peak $\dot{M}O_{2}$ value following *P*_LOE_. Therefore, whilst the 25°C 30-day snapper appeared to maintain similar *P*_crit_ and *P*_LOE_ as the 21°C control group, O_2_ debt differed between treatments. Most likely the 30-day 25°C group elevated anaerobic metabolism to withstand greater O_2_ debt than the control group. It should be noted that another limitation of using oxygen uptake as a proxy for ATP turnover is its inability to account for anaerobic metabolism. In addition, OXPHOS was assessed using carbohydrate derived substrates only, and the synergetic implement of NADH derived from beta-oxidation was omitted. Examining anaerobic pathways as well as the potential additive effect of beta oxidation towards ATP production would therefore probably provide considerable insight into the ability of 25°C 30-day snapper to enlarge their oxygen debt following *P*_LOE_.

An enlargement of relative ventricular mass in response to the MHW scenario, by 19% following 30-days exposure, may have assisted snapper in enhancing both their MMR and MS. Indeed, the ability of fish to maintain MMR across a range of temperatures is dependent on factors of oxygen delivery and uptake, including cardiac output and blood oxygen carrying capacity (Albers et al., 1983; Gamperl & Farrell, 2004; Claësson et al., 2016). Whilst a larger heart suggests the possibility of an increased cardiac output for these groups, direct measures of cardiac function were not taken and should be explored in future research to confirm this. The relative liver mass also differed between treatment groups, but this included a significant decline between the first and second 21°C controls, which highlighted an effect of time in captivity, possibly from the diet change from that in the wild to the commercial fish feed pellets. This may also be due to the seasonal growth differences and storage pattern adopted over time by snapper, where changes to body composition, energy storage, and relative organ mass have been shown to vary between seasons (Francis, 1994b; Sim-Smith et al., 2013a; Wellenreuther et al., 2019; Flikac et al., 2020; Cook et al., 2021). No further conclusions were therefore drawn from liver mass samples as it was impossible to disentangle the effects of temperature from time in captivity for this one measurement variable.

### Are snapper resilient to climate change stressors?

This study aligns with many others that demonstrate the resilience of snapper from many physiological stressors, including exposure to turbidity (Cumming & Herbert, 2016), high carbon dioxide (McMahon et al., 2019; 2020), hypoxia (Cook et al., 2011; Cook & Herbert, 2012; Cook et al., 2013), angling stress (McArley & Herbert, 2014), low temperatures (Wolfe et al., 2020), and seasonal temperature variability (Cook et al., 2021). This research therefore provides additional evidence in support of the notion proposed of Parsons et al. (2014) that regional temperature increases in New Zealand may lead to more productive and abundant snapper in the SNA1 stock area. This may even occur over a greater geographical range, considering the strong positive association between temperature and juvenile snapper growth (Francis, 1993; Francis, 1994b). The impact of climate change will almost certainly vary according to life stage but early life stages of fish are notoriously sensitive to environmental change and represent a large knowledge gap for snapper (Parsons et al., 2014).

This study sought to address the potential synergistic effect of viable thermal stress and hypoxia tolerance on snapper. A particularly impressive finding of this study, building on the picture of snapper resilience, was the lack of change in hypoxia tolerance between fish acclimated to 21°C and 25°C (30-days). Indeed, whilst metabolism increased as expected under warmer 25°C conditions, it did not coincide with an expected decline in hypoxia tolerance. Additionally, the current *P*_crit_ values of 5.4 and 5.3 kPa (ca. 25%–26% air saturation), following acclimation to 21°C and 25°C respectively, are essentially equivalent to the 5.3–5.8 kPa *P*_crit_ level of snapper at 18°C in other studies (Cook et al., 2011; Cook & Herbert, 2012). Thus, snapper *P*_crit_ appears to remain surprisingly steady from 18°C to 25°C (Cook et al., 2011), suggesting a very large window of optimum temperature for hypoxia tolerance in this species. This was unexpected, as past research commonly shows an increase in fish *P*_crit_ with temperature (Fernandes & Rantin, 1989; Schurmann & Steffensen, 1997; Rogers et al., 2016). For example, the *P*_crit_ of the Atlantic cod (*Gadus morhua*) increased dramatically from 3.4 kPa at 5°C to 6.4 kPa at 15°C (Schurmann & Steffensen, 1997). Some studies argue that *P*_LOE_ is a better indicator of hypoxia tolerance than *P*_crit_ (Wood, 2018) but the *P*_LOE_ of snapper also recovered and was not significantly different between the 21°C and 30-day 25°C group (i.e., 2.8 kPa and 3.1 kPa respectively). Whilst snapper appear robust to the synergistic effects of thermal challenge and hypoxia, it should be noted that their *P*_LOE_ is close to the *P*O_2_ at which they behaviourally avoid hypoxia at 18°C (3.1 kPa). The *P*_LOE_ for snapper at 18°C is currently unknown but the lack of change in both *P*_crit_ from 18°C to 25°C (Cook et al., 2011) and *P*_LOE_ from 21°C to 25°C (current study data) suggests that *P*_LOE_ at 18°C may be similar to the values obtained in the current study. Thus, the point where snapper behaviourally act to avoid hypoxia is potentially only just above their *P*_LOE_, leaving little margin of safety for survival if severe hypoxia is ever experienced. As snapper are only considered to be moderately hypoxia tolerant (Cook & Herbert, 2012) and employ a ‘dangerous’ hypoxia avoidance strategy (Cook et al., 2011), this species may have to rely on anaerobic metabolism and always be in a state of stress at the point of avoiding extreme low O_2_ (Cook & Herbert, 2012).

## Conclusion

This study demonstrates that the whole body and subcellular metabolic systems of snapper from the SNA1 stock are resilient to thermal MHW conditions, and this resilience is retained by snapper during simultaneous low O_2_ (hypoxia) exposures. Various measures of metabolic performance were employed in this study across multiple levels of biological organisation, including a variety of whole body O_2_ uptake and heart mitochondrial OXPHOS measures, which either remained the same or improved following exposure to an extreme MHW scenario (25°C) for north-eastern New Zealand. When hypoxia was added as a secondary stressor to the MHW challenge, measures of acute hypoxia tolerance (e.g., *P*_crit_, *P*_LOE_ thresholds, etc.) also recovered over a period of 10- or 30-days to levels exhibited by fish at 21°C. The current research therefore aligns strongly with other studies describing the physiological resilience of snapper to a wide variety of stressors, including high temperatures, hypoxia, CO_2_, and turbidity (Cook et al., 2011; Cook & Herbert, 2012; Cook et al., 2013; McArley & Herbert, 2014; Cumming & Herbert, 2016; McMahon et al., 2019; 2020; Cook et al., 2021). The notion proposed by Parsons et al. (2014) is thus supported by the current study: “Regional temperature increases in New Zealand could feasibly lead to more productive and abundant snapper over a greater geographical range”. In contrast to the usual doomsday depiction, SNA1 New Zealand snapper may be one of the front running species in the emerging era of climate change, at least in terms of metabolic physiology (Watson, 2018). Whether the same is true for other keystone species warrants further investigation and remains to be seen. It is important to note that high temperatures and hypoxia are not the only stressors faced by fish in New Zealand. Applying high temperature with other anthropogenic pressures, such as turbidity, nutrient loading, and reduced prey availability, could potentially reduce the resilience of snapper and other fish species when layered simultaneously. Future research is required to resolve these unknowns and to consider the wider aspects of climate change across a broader range of species.

## Figures and Tables

**Table:** 

**ΔΨm**	Proton motive force
**ANOVA**	Analysis of variance
**Asc**	Ascorbate
**ADP**	Adenosine diphosphate
**ATP**	Adenosine triphosphate
**AZ**	Sodium azide
**CI**	Complex I
**CII**	Complex II
**CIII**	Complex III
**CCCP**	Carbonyl cyanide m-chloro phenyl hydrazone
**CCO**	Complex IV (cytochrome c oxidase)
**CO**_**2**_	Carbon dioxide
**[Hb]**	Concentration of haemoglobin
**H**_**2**_ **O**	Water
**H**_**2**_ **O**_**2**_	Hydrogen peroxide
**Hct**	Haematocrit
**JO**_**2**_	Mass-specific mitochondrial respiration flux
**MCH**	Mean corpuscular haemoglobin
**MHW**	Marine heatwave
**MMR**	Maximum metabolic rate
***Ṁ*O**_**2**_	Mass specific rate of oxygen consumption
**MS**	Metabolic aerobic scope
**O**_**2**_	Molecular Oxygen
**O2k**	Oroboros Oxygraph-2k respirometer
**OCLTT**	Oxygen- and capacity-limited thermal tolerance theory
**OXPHOS**	Oxidative phosphorylation
***P*50**_**mito**_	Mitochondrial oxygen affinity
***P***_**crit**_	Critical oxygen pressure
***P***_**LOE**_	Oxygen pressure at loss of equilibrium
***P*O**_**2**_	Partial pressure of oxygen
**RCR**	Respiratory control ratio
**Safr**	Safranin-O
**SE**	Standard error
**SMR**	Standard metabolic rate
**SST**	Sea surface temperature
**TMPD**	N,N,N′,N′-tetramethyl-p-phenylenediamine

## Data Availability

The raw data supporting the conclusion of this article will be made available by the authors, without undue reservation.
